# Stokes settling and particle-laden plumes: implications for deep-sea mining and volcanic eruption plumes

**DOI:** 10.1098/rsta.2019.0532

**Published:** 2020-08-03

**Authors:** Nicola Mingotti, Andrew W. Woods

**Affiliations:** BP Institute for Multiphase Flow, University of Cambridge, Cambridge, UK

**Keywords:** particle settling, deep-sea mining, eruption

## Abstract

Turbulent buoyant plumes moving through density stratified environments transport large volumes of fluid vertically. Eventually, the fluid reaches its neutral buoyancy level at which it intrudes into the environment. For single-phase plume, the well-known theory of Morton, Taylor and Turner (Morton BR, Taylor GI, Turner JS. 1956 Turbulent gravitational convection from maintained and instantaneous sources. *Proc. R. Soc. A* **234**, 1–23. (doi:10.1098/rspa.1956.0011)) describes the height of the intrusion with great accuracy. However, in multiphase plumes, such as descending particle plumes formed from the surface vessel during deep-sea mining operations, or ascending volcanic plumes, consisting of hot gas and dense ash particles, the sedimentation of particles can change the buoyancy of the fluid very significantly. Even if the plume speed far exceeds the sedimentation speed, the ultimate intrusion height of the fluid may be significantly affected by particle sedimentation. We explore this process, illustrating the phenomena with a series of analogue experiments and some simple modelling, and we discuss the applications in helping to quantify some environmental impacts of deep-sea mining and in helping to assess the eruption conditions leading to the formation of large laterally spreading ash clouds in the atmosphere.

This article is part of the theme issue ‘Stokes at 200 (part 2)’.

## 1. Introduction

Turbulent plumes are produced by the release of buoyant fluid from a localized source. In a density stratified environment, the net buoyancy of the plume gradually decreases with height until a neutral buoyancy height is eventually reached, at which the ascent is arrested and the fluid spreads laterally into the environment. The dynamics of such steady turbulent plumes was described in detail by Morton *et al.* [1]. This model provides invaluable scaling laws for the characteristic speed, *u*, and the total height of rise of the plume in a stratified ambient, *H*, in terms of (a) the source buoyancy flux,

1.1 $$B_{0}=g_{0}^{\prime }Q_{0},$$

where *g*′_0_ is the reduced buoyancy and *Q*_0_ the volume flux at the source, and (b) the ambient stratification, as measured by the Brunt–Väisälä frequency *N*, where

1.2 $$N^{2}=-\frac{g}{\rho_{0}}\frac{\text{d}\rho }{\text{d}z}.$$

The height of rise of the plume is given by the expression (cf. [1–3])

1.3 $$H=5\;\pi^{-1/4}B_{0}^{1/4}N^{-3/4},$$

and the height of the intrusion of the fluid in a single-phase plume is given by [3]

1.4 $$H_{i}=4\;\pi^{-1/4}B_{0}^{1/4}N^{-3/4}.$$

If the plume fluid contains small, heavy particles with a settling speed *v*_*s*_ which is much smaller than the characteristic speed of the plume, *u*, as given by the relation

1.5 $$v_{s}\ll u\approx 3\;B_{0}^{1/4}N^{1/4},$$

then to leading order the particles are carried by the plume, and we expect it to behave analogously to a single-phase plume up to the neutral buoyancy height. However, as the plume fluid begins to spread radially at this level, the particles gradually fall out of the cloud and the residual fluid, therefore, becomes less dense. The particles may contribute a significant fraction of the buoyancy of the fluid supplied by the plume to the intrusion and so sedimentation can then lead to a significant change in the neutral buoyancy height of the intrusion. Assessment of these effects forms a major focus of this work.

A key factor we need to understand in order to model the effects of particle sedimentation is the effective buoyancy of the fluid entrained between the source and the top of the plume. The buoyancy of the intruding fluid multiplied by its volume flux, $g_{f_{H}}^{\prime }Q_{H}$, is given by the combination of the buoyancy of the source fluid multiplied by the volume flux of the source fluid, $g_{f_{0}}^{\prime }Q_{0}$, together with the average buoyancy of the entrained fluid multiplied by the volume flux of the entrained fluid, $g_{f_{e}}^{\prime }Q_{e}$. Typically *Q*_*e*_ ≫ *Q*_0_, and so henceforth we set *Q*_*e*_ = *Q*_*H*_ and obtain

1.6 $$g_{f_{H}}^{\prime }Q_{H}=g_{f_{0}}^{\prime }Q_{0}+g_{f_{e}}^{\prime }Q_{H}.$$

The average buoyancy of the entrained fluid relative to the ambient fluid at the source is given by the expression

1.7 $$g_{f_{e}}^{\prime }=\frac{1}{Q_{H}}\int_{0}^{H}Nz\frac{\text{d}Q}{\text{d}z}\;\text{d}z,$$

In order to calculate the integral in equation (1.7), we require an expression for *dQ*/*dz*. In the classical theory of turbulent buoyant plumes, the mass, *Q* = *πq*, momentum, *M* = *πm*, and buoyancy, *B* = *πb*, fluxes evolve according to the following conservation equations:

1.8 $$\frac{\text{d}q}{\text{d}z}=2\alpha m^{1/2},\;m\frac{\text{d}m}{\text{d}z}=bq\;\text{and}\;\frac{\text{d}b}{\text{d}z}=-N^{2}q,$$

where *Q*, *M* and *B* are given by the following expressions [1]:

1.9 $$Q=2\pi \int_{0}^{\infty }r\rho u\;\text{d}r,\;M=2\pi \int_{0}^{\infty }r\rho u^{2}\;\text{d}r\;\text{and}\;B=2\pi \int_{0}^{\infty }rg^{\prime }u\;\text{d}r,$$

in which *r* is the radial distance from the plume axis, *ρ* is density, *u* the speed of the plume fluid, and *g*′ the reduced gravity of the plume fluid. In the case of an unstratified environment, there is a self-similar relation for the volume flux *Q*(*z*),

1.10 $$Q(z)=0.1\;B_{0}^{1/3}z^{5/3},$$

where the coefficient 0.1 is determined empirically and is related to the entrainment coefficient *α* [1]. In a stratified environment, however, the volume flux *Q*(*z*) closely follows equation (1.10) in the region below the neutral buoyancy height, and so equation (1.10) provides an approximation for evaluating *dQ*/*dz* in the integral equation (1.7). This leads to the approximate result

1.11 $$g_{f_{e}}^{\prime }\approx \frac{5}{8}N^{2}H,$$

in which *H* is the maximum height reached by the plume (see equation (1.3)). Full numerical solution in fact leads to the value

1.12 $$g_{f_{e}}^{\prime }=0.58N^{2}H.$$

We deduce that the average buoyancy of the entrained fluid is approximately equivalent to that of the ambient fluid at a height 0.58 H above the source in a stratified layer. As a result, we expect that in the event that all particles sediment from the intrusion, the buoyancy of the intruding fluid will be

1.13 $$g_{f_{H}}^{\prime }=g_{f_{0}}^{\prime }\frac{Q_{0}}{Q_{H}}+0.58N^{2}H,$$

where $g_{f_{0}}^{\prime }$ corresponds to the buoyancy of the source fluid relative to that of the ambient fluid at the level of the source, *Q*_0_ is the source volume flux and *Q*_*H*_ is the volume flux at the top of the plume. In this paper, we consider descending plumes (which relate to deep-sea mining operations) in which the particles add to the total buoyancy of the plume, and also ascending plumes (with relevance to volcanic eruptions) in which the particles reduce the effective buoyancy of the plume. In both cases, we will use the simplified relation 1.13 for the buoyancy of the particle-free intrusion fluid to estimate the height of the intrusion following sedimentation.

**Figure 1.**
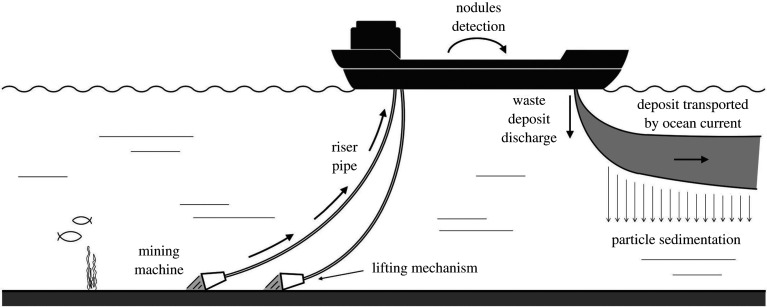
Schematic of a particle plume developing during a deep sea mining process, as particles are released from a surface ship into the ocean. The depth of the intrusion of the water within the particle plume is key for assessing the fate of any minerals dissolved into the water, while the controls on the migration of the particles through the water column are key for assessing the area over which the particles may be dispersed.

In deep-sea mining, particles of waste material may be released from a ship, near the surface (figure 1) [4,5]. These may then sink through the ocean to form a particle-driven plume [4,6]. Some of the minerals in this plume may dissolve into the plume water, leading to a possible environmental impact associated with the plume water as well as the particles. We illustrate how the approximate depth of this plume water as it intrudes into the stratified water column may be predicted in the case that the particles are sufficiently small that their settling speed is much smaller than the convective speed of the plume, so that they are carried with the plume (cf. [3,7]). We also show that the subsequent motion of the particles is dominated by their gravitational settling through the water column. In contrast, if the particle plume sinks through a two-layer stratified ambient, the plume fluid may intrude at the interface, while the particles settle through the interface and gradually converge to form a new secondary plume in the lower layer. If, as in the ocean, this lower layer is weakly stratified, then our experiments suggest that this secondary plume may be arrested at a greater depth, forming a second intrusion, while the particles will again gradually settle out below this level.

In a volcanic eruption, a dense fountain of heavy particles and relatively buoyant fluid issues from a volcanic vent. If the mass eruption rate is sufficiently small, or the eruption velocity sufficiently large, then this ascending mixture may entrain and heat up sufficient air to become buoyant relative to the surrounding air [8,9]. The majority of the fine-grained fraction of the erupted material then rises as a buoyant plume until it reaches its neutral buoyancy height [8,9], while some of the larger particles may settle from the plume once their fall speed becomes comparable to that of the ascending plume. In highly explosive eruptions, the majority of the material is in fact very fine grained [9], and so has a small fall speed relative to the speed of the plume. Hence, this material rises with the plume to the neutral height [10], and we explore this limit in the present work, assuming all particles are carried upwards with the plume. However, as the mixture begins to spread laterally at this neutral height, the particles gradually separate from the ash-laden air, falling back through the environment towards the ground [11–14].

**Figure 2.**
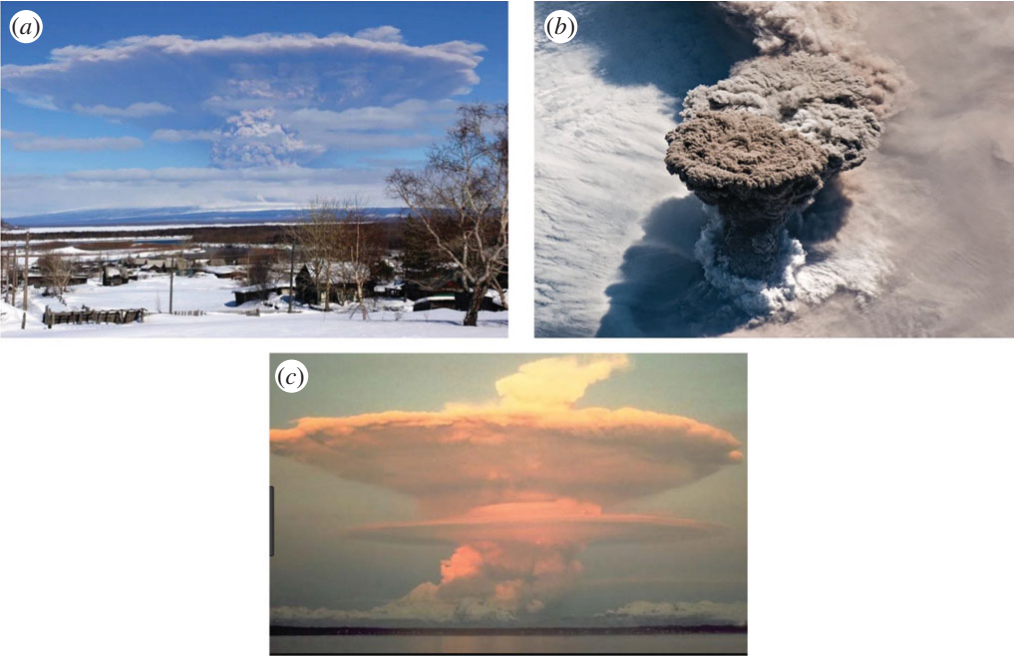
Eruption clouds from (*a*) Mt Shiveluch, Kamchatka, 2007 (courtesy Demyanchuk, Yuri); (*b*) Raikoke, Kuril Islands, 2019 (Joshua Stevens, NASA Earth Observatory); and (*c*) Mt Redoubt, Alaska, 1991. The images show that the development of the laterally spreading neutral cloud has an asymmetric shape, as the cloud seems to have a relatively flat upper surface and an upward sloping lower surface. We propose this is related to the sedimentation of particles, which descend about the rising eruption column, and which reduce the density of the remaining hot air and fine ash in the spreading cloud. (Online version in colour.)

In nature, it is difficult to observe all the processes in detail during an intense explosive eruption. However, observation of the neutral or umbrella clouds at a number of eruptions suggest that the laterally spreading neutral cloud is somewhat asymmetric relative to a horizontal plane; there is an upward drift of the cloud as it spreads out, as may be seen in the images from the eruption at Mt Shiveluch (2007) and Mt Redoubt (1991) [15], and consistent with the satellite image of the neutral cloud at the eruption of Raikoke (2019) (figure 2). Improving our understanding of the controls on the height and dynamics of these intrusions is valuable for the assessment of the hazards of volcanic ash, particularly for air-traffic safety. In this context, it is important to note that there are in fact several very detailed numerical models of the eruption column and umbrella cloud which have been developed to account for effects of buoyancy-driven spreading of the umbrella cloud [16–18] and in some cases the effect of wind on the eruption column and neutral cloud [12,13,19]. Some of these models describe the sedimentation of particles from the flow, but there has been less focus on the impact of the change in the buoyancy and hence height of the spreading intrusion associated with sedimentation. Even if a fraction of the particles sediments from the flow as the neutral cloud spreads from the vertical plume, there may be a change in the buoyancy and hence height of the intrusion, and investigation of this process forms the focus of this work. Since the air–particle mixture at the neutral height has the same bulk density as the surrounding air, the air in the cloud is in fact warmer than the surrounding atmosphere [20] and so sedimentation of the particles can lead to a gradual increase in height of the neutral cloud. With particle fall speeds in the range of 0.01–1 m s^−1^, and intrusion speeds of order 10–100 m s^−1^, the intrusion may spread a horizontal distance of order 10–1000 km as the particles settle through a height of 1–5 km, comparable to the thickness of the intrusion. We might therefore expect to see some change in elevation of the neutral cloud resulting from such sedimentation. The approach in this paper is to develop a very simplified model of the sedimentation process in order to explore the physical controls on the process, rather than develop a detailed numerical simulation. In the future, it would be interesting to develop the present work to include some of the additional details and complexities.

As mentioned above, these fluid–particle processes are complex, and so there is value in developing small-scale idealized laboratory experiments with mixtures of particles in fresh or saline aqueous solution, to explore some of the leading order controls on the flow. Furthermore, it is possible to make high-resolution digital images of the evolution of such experiments, and using light attenuation techniques to quantify the speed, concentration and fate of the different components of the system [3]. Such experimental modelling, when combined with scaling analysis, can also provide insight into some of the controls on the larger scale geophysical system. There have been a number of experimental papers examining the particle re-entrainment process in a turbulent buoyant plume, many of which have considered the phenomena in an unstratified environment [21–23]. Additionally, some papers have investigated the case of particle plumes rising through a stratified ambient (e.g. [10,24,25]), and introduced a theoretical framework which has recently been further developed by Apsely & Lane-Serff [26]. Two recent experimental studies have considered the dynamics of particle plumes in a stratified environment [27,28], focusing on the case of an ascending plume in which the particle load reduces the initial buoyancy of the plume. The main emphasis of these papers was related to the height of rise of the plume, as well as aspects of the re-entrainment of the particles into the plume. However, they did not explore the change in buoyancy of the radially intruding fluid supplied by the plume as a result of the sedimentation of the particles. In the case that the fall speed of the particles is much smaller than the rise speed of the plume, there is a separation of time scales between the sedimentation of particles from the intruding fluid and the adjustment of the height of the intruding fluid to its new, particle-free neutral buoyancy height. The focus in this paper is this adjustment to the neutral buoyancy height of the spreading intrusion in this limit, which thereby provides a complement to the earlier studies listed above.

We note that the descending particle plume experiments in a uniformly stratified ambient which we describe below have been presented by Mingotti & Woods [3], but these provide a useful reference, and inform aspects of the behaviour of particle plumes in a two-layer ambient as considered in the present work. They also help in guiding the interpretation of the experiments we report herein on fresh water plumes laden with particles as they rise through a stratified environment. These latter experiments model aspects of buoyant volcanic eruption columns and especially the controls on the height of the intrusion. There have been some earlier experiments exploring such particle-laden, fresh water plumes, but they have primarily focused on some of the processes of fallout and re-entrainment of particles, using a uniform density ambient fluid [21,23,29,30]. Here, we explore the interaction of the stratification in the ambient with the particle-laden buoyant plume, and the role of the particle–fluid separation in influencing the intrusion height of the fluid above the plume.

## 2. Experiments

**Figure 3.**
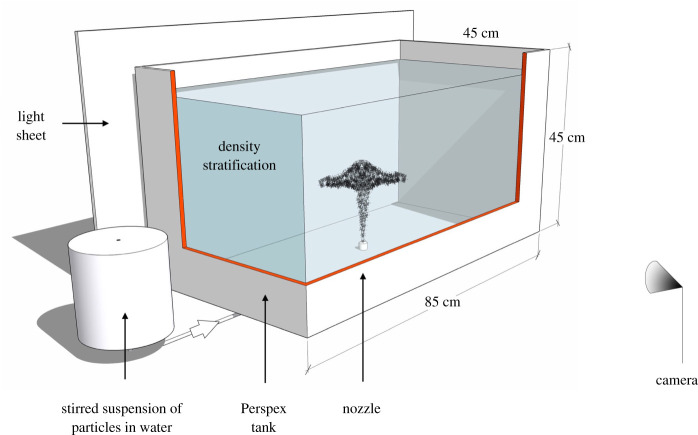
Experimental set-up. The source nozzle was placed either at the top or the base of the tank to model pure particle plumes or buoyant plumes of fresh water laden with particles. (Online version in colour.)

We present two key types of experiment in which we release combinations of fresh or saline fluid, laden with particles, from a localized source into a density stratified environment. The experimental apparatus consists of a rectangular, Perspex tank of dimension 85 × 45 × 45 cm, which is filled with a linearly stratified solution of salt obtained using the double-bucket method (cf. [31], figure 3). A stirred reservoir containing particle-laden water or particle-laden saline solution is located beside the tank; the temperature of the fluid in this reservoir equals that of the ambient fluid in the tank. During each experiment, a constant flux of particle-laden fluid is drawn from the reservoir and supplied to the tank using a peristaltic pump. Silicon carbide particles of a density 3.22 g cm^−3^ and sizes ranging between 20 and 150 μm have been used in this study: these particles have fall speeds *v*_*s*_ of order 0.1–1 cm s^−1^ in water (tables 1–3). The particle-laden fluid is supplied through a localized nozzle of an internal diameter 1 mm, which is either located at the top of the tank in the case of a pure particle plume (see §3), or at the base of the tank in the case of a buoyant plume of fresh water, laden with particles, which provides a simplified model of the buoyant part of a volcanic eruption column (see §4). On entering the tank, the particle-laden fluid forms a plume. For buoyancy fluxes of order *B*_0_ ≈ 10^−6^ m^4^ s^−3^ (tables 1–3), the time scale for a change in the mean plume flow speed is of order $t_{f}={\left(\text{d}u/\text{d}z\right)}^{-1}=B_{0}^{-1/3}z^{4/3}\approx 4.5\text{–}11.5\;\text{s}$ at a distance z=10–20 cm from the source: this is much larger than the time scale for the particle speed to adjust to the plume flow speed, *t*_*p*_ = *ρ*_*p*_ *d*^2^/18 μg ≈ 10^−3^ s, where *ρ*_*p*_ is the particle density, *d* is its diameter and μ is the fluid viscosity. Consequently, we expect the silicon carbide particles to move with the flow in our experiments. This is consistent with the expected behaviour of the particles in the full-scale flows. In the ocean, the buoyancy flux of deep-sea mining plumes may be of order $1\text{–}100\;{\text{m}}^{4}\;{\text{s}}^{-3}$, and so we calculate that at a depth of about 10–20 m below the surface the time scale for a change in the mean flow speed will be of order $t_{f}\approx 10\text{–}20\;\text{s}$. For particles of size *d* ≈ 1 mm, the Stokes flow response time is of order *t*_*p*_ ≈ 0.1 s, and so we expect these particles to behave as per our experiments. In volcanic ash plumes, the time scale of the flow is of order $t_{f}\approx 10\text{–}100\;\text{s}$, depending on the height and size of the plume. For particles sizes of order *d* ≈ 1 mm, the turbulent drag on the particle has a time scale of order *cρ*_*a*_ *u*/*dρ*_*p*_. For a typical drag coefficient *c* ≈ 0.1, we obtain that the turbulent drag on the particle has value of about 1–10 s, so that ash of this size will tend to move with the air. However, ash smaller than about 0.1 mm will fall at the Stokes settling speed and have a faster response time, so we expect ash smaller than about 1 mm to move with the plume.

**Table 1.** Conditions of the deep-sea mining, pure particle plume experiments depicted in figures 5–6. *Q*_0_ (m^3^ s^−1^) denotes the plume volume flux at the source; *C*_0_ is the concentration of particles in the source fluid; *g*′_0_ (m s^−2^) is the bulk reduced gravity of the source particle-laden fluid; *B*_0_ (m^4^ s^−3^) is the buoyancy flux at the source; *v*_*s*_ (m s^−1^) is the mean particle fall speed; *N* (1 s^−1^) is the Brunt–Väisälä buoyancy frequency of the ambient fluid in the tank; and Re is the Reynolds number at the source.

exp.	*Q*_0_ × 10^−6^	*C*_0_	*g*′_0_	*B*_0_ × 10^−6^	*v*_*s*_ × 10^−3^	*N*	Re
a	1.00	0.092	2.00	2.00	1.03	0.73	2546
b	1.50	0.092	2.00	3.00	1.61	0.73	3820
c	1.00	0.092	2.00	2.00	1.61	0.73	2546
d	0.50	0.092	2.00	1.00	1.61	0.73	1273
e	1.00	0.092	2.00	2.00	2.39	1.46	2546
f	0.50	0.184	4.00	2.00	2.39	0.73	2546
g	1.00	0.092	2.00	2.00	2.39	0.37	2546
h	1.50	0.092	2.00	3.00	4.80	0.73	3820
i	1.00	0.092	2.00	2.00	4.80	0.73	2546
j	1.00	0.092	2.00	2.00	6.80	0.73	2546
k	1.00	0.092	2.00	2.00	13.58	1.62	2546
l	1.00	0.092	2.00	2.00	54.30	0.97	2546

**Table 2.** Conditions of the two-layer particle plume experiments depicted in figure 7. In these experiments, the height of the upper layer was 12 cm, and the uniform density of the fluid in the upper layer was 1 g cm^−3^. *N* (1 s^−1^) is the Brunt–Väisälä buoyancy frequency of the ambient fluid in the lower layer.

exp.	*Q*_0_ × 10^−6^	*C*_0_	*g*′_0_	*B*_0_ × 10^−6^	*v*_*s*_ × 10^−3^	*N*	Re
m	1.00	0.092	2.00	2.00	1.61	0.246	2546
n	1.00	0.092	2.00	2.00	1.61	0.318	2546
o	1.00	0.092	2.00	2.00	1.61	0.492	2546

**Table 3.** Conditions of the volcanic plume experiments depicted in figures 9 and 11. Here, *g*′_0_ (m s^−2^) is the bulk reduced gravity of the source particle-laden fluid, while *g*′_*p*_ and *g*′_*f*_ denote the reduced gravity associated with the particles and the plume fluid respectively (see equation (4.1)).

exp.	*Q*_0_ × 10^−6^	*C*_0_	*g*′_0_	*g*′_*f*_	*g*′_*p*_	*B*_0_ × 10^−6^	*v*_*s*_ × 10^−3^	*N*	Re
p	4.00	0.050	0.725	1.812	1.087	2.90	1.61	0.729	4529
q	4.00	0.042	0.725	1.631	0.906	2.90	1.61	0.729	4529
r	4.00	0.033	0.725	1.450	0.725	2.90	1.61	0.729	4529
s	4.00	0.025	0.725	1.269	0.544	2.90	1.61	0.729	4529
t	4.00	0.017	0.725	1.087	0.362	2.90	1.61	0.729	4529
u	4.00	0.008	0.725	0.906	0.181	2.90	1.61	0.729	4529

To compare the behaviour of each particle plume listed in tables 1–3 to that of a single-phase plume, a small amount of saline aqueous solution containing blue dye was supplied to the tank through the same nozzle and with the same total buoyancy flux described above (figures 6 and 9). The density of the blue fluid was equal to the bulk density of the particle-laden fluid in the stirred reservoir. For each experiment, the height of the intrusion of the blue fluid was found to be in good agreement with the theoretical prediction for a turbulent single-phase plume (see equation (1.3)), assuming an entrainment coefficient *α* = 0.11 ± 0.02 [1]. Each experiment was recorded using a digital Nikon D5300 camera, taking images at 60 frames per second. A light sheet was used to provide a uniform source of lighting for image analysis purposes (further details may be found in Mingotti & Woods [3]).

## 3. Deep-sea mining: pure particle plumes

**Figure 4.**
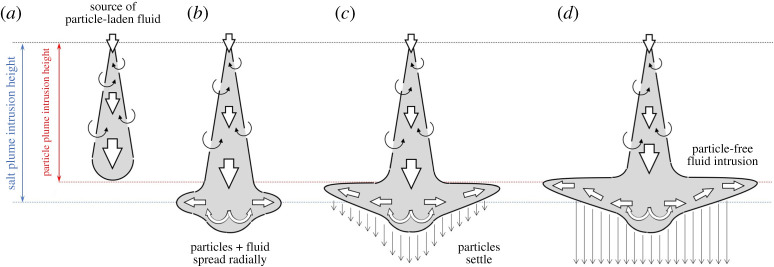
Cartoon illustrating the behaviour of a pure particle plume descending through a stratified ambient, which has relevance for deep-sea mining operations (see §3). (*a*) Heavy particles are released at the surface and form a turbulent descending plume which entrains ambient fluid; (*b*) upon reaching the neutral buoyancy level, the mixture of particles and plume fluid spreads radially; (*c*) particles separate from the intruding fluid and settle; and (*d*) the remaining fluid is now depleted of particles and so it rises to its new neutral buoyancy level, forming a shallower intrusion. (Online version in colour.)

In the case of a pure particle plume, the buoyancy of the dense particles drives the flow downwards through a stratified ambient (figure 4*a*). The flow is turbulent and the particles and fluid descend to the level at which the bulk speed of the flow falls to zero. After an initial overshoot, the flow begins to spread laterally at its neutral buoyancy level (figure 4*b*). There is then a zone of fluid–particle separation (figure 4*c*), with particles falling down while the residual fluid becomes more buoyant (figure 4*d*). We first explore how a particle-driven plume moves downwards through a continuously stratified ambient fluid, as a model of a particle plume produced during deep-sea mining operations. We then explore the case in which there is an upper well-mixed layer underlain by a continuous but weak stratification in the lower zone, as a model of the shallow upper layer and deep weakly stratified lower ocean, representative of the stratification in the Pacific Ocean [6].

**Figure 5.**
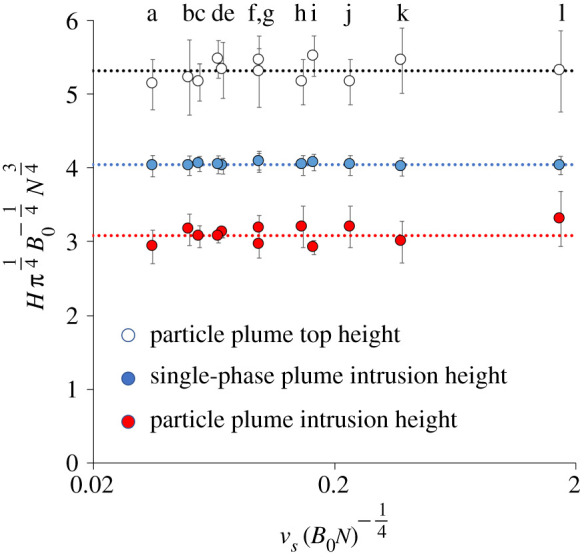
Height of the intrusion in a particle plume (red) and a normal saline plume (blue) relative to the maximum initial height of the plume (open circles) with the same buoyancy flux (experiments a–l, table 1.) (Online version in colour.)

With a uniform stratification, a particle-driven plume is gradually arrested by the mixing and entrainment of the ambient fluid through which it descends. Based on classical plume theory [1], the initial distance *H* travelled by the plume on becoming arrested by the stratification depends on the stratification intensity, as measured by the Brunt–Väisälä frequency, *N* and the buoyancy flux, *B* (see equation (1.3)). On reaching this height, some of the particles continue to descend, while the remaining fluid, which is a mixture of the source fluid and the fluid entrained from the environment, rises back to the neutral buoyancy height. Using the simplified relation 1.13 based on the classical plume theory discussed in §1, we estimate that in a turbulent plume, the fluid at the maximum height of rise of the plume has a buoyancy flux of which approximately 3/8 arises from the buoyancy of the source fluid and 5/8 is associated with the entrainment of ambient fluid as it moves down through the water column. Therefore, if all the particles separate from the flow and fallout, the density of the remaining fluid is equivalent to that of the fluid a distance 5 *H*/8 below the source. Mingotti & Woods [3] demonstrated that this is the case by measuring the maximum height and the intrusion height of a series of particle plumes in which the ratio of the fall speed to the plume speed is varied but remains small (figure 5).

For deep-sea mining, this is a key result, since it demonstrates that the fluid carried down from the surface in a particle plume moving through the stratified upper water column may intrude at a depth of about

3.1 $$H_{i}=0.58H=2.9\;\pi^{-1/4}B_{0}^{1/4}N^{-3/4}.$$

**Figure 6.**
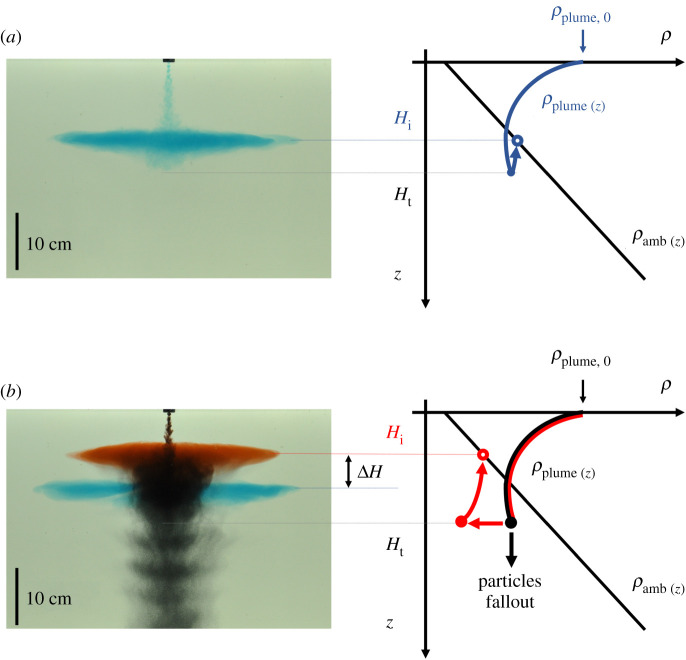
Comparison of (*a*) a saline plume and (*b*) a particle plume descending from the top of the reservoir (experiment i, see table 1). Once the plume reaches the neutral density level, the particles sediment, leading to a large drop in the buoyancy, and hence the ascent of the neutral cloud to a point higher in the water column. This is illustrated by the red dyed fluid in the particle plume, which intrudes at a shallower depth than the blue dyed fluid in the saline plume in (*b*). (Online version in colour.)

The physical process of entrainment and mixing as the plume descends to the neutral buoyancy level, and the implications of particle separation on the intrusion height of a multiphase plume are illustrated in figure 6.

In order to put these results into context, we first consider the case in which the upper layer above the thermocline is stratified. With typical values of the stratification in the upper ocean *N* = 0.001 s^−1^ and particle fluxes in the range 0.1–10.0 m^3^ s^−1^, then assuming the particle density is about 2.5 times that of the water, the buoyancy flux would be $B_{0}=1.0\text{–}100.0\;{\text{m}}^{4}\;{\text{s}}^{-3}$, and we estimate an intrusion height in the range 10–100 m. This is very shallow; very fine particles and soluble minerals may remain in the water in this intrusion as the coarser particles settle to deeper waters in the ocean.

**Figure 7.**
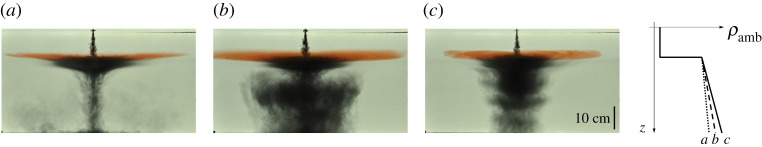
Illustration of a particle plume moving through a well-mixed zone underlain by a weakly stratified zone (experiments m-o, see table 2). (*a*) The weakly stratified case, in which the particles are drawn together on leaving the interfacial intrusion, and form a new particle plume; (*b*) The intermediate stratified case, in which the particles settle from the interfacial intrusion to form a nascent plume, which is then arrested by the stratification, forming a large intruding layer; (*c*) The more strongly stratified case in which the particles are unable to establish a particle plume prior to the convective downflow being arrested by the stratification. As a result, particles settle through the liquid forming a series of circulation patterns (cf. [3]). The photographs were captured at times (*a*) 45s, (*b*) 57s and (*c*) 59s after the beginning of each experiment. (Online version in colour.)

In the second case, we consider that the upper layer of the ocean may be well-mixed owing to surface cooling and wind stresses, and so the particle plume may reach the lower boundary of the mixed layer. If there is a density jump here, the plume may entrain a small amount of the denser fluid from below the thermocline (cf. [7]) and then intrude at the thermocline. In this case, as the particles settle from the spreading intrusion, the fluid will tend to remain at the thermocline as it will have a density intermediate to the two layers, as seen in the experiments depicted in figure 7. However, as the particles settle from the intrusion into the lower layer, the particles may drive a convective downflow. Typically the stratification in the deep ocean is very weak, and so this convective downflow may form a weak descending plume, initially drawing in the fluid–particle mix settling from the intrusion. However, deeper below the interface, as the entrainment begins to dominate, the plume will start to grow in radius.

If the lower layer has a sufficiently weak stratification, then the plume will tend to move downwards through the lower layer and reach the base of the system, as shown in figure 7a. However, for larger stratification, as the plume entrains the shallower and less dense fluid, it may become arrested by the stratification. This might lead to a second intrusion in the deeper layer, as illustrated in figure 7*b* from which the particles will then sediment through the water column. With even stronger stratification, the plume will not fully develop, but a cylinder of descending particles will form, with a series of small intrusion-type features at the periphery [3]. These features migrate up through the particle column as wave-type structures, through a combination of particle settling and local convective recirculation at the edge of the column, and so are only transient features; the main control on the particle transport in this region is the particle fall speed [3].

## 4. Volcanic plumes

**Figure 8.**
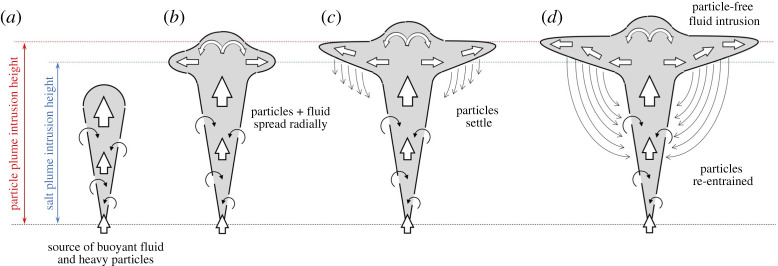
Cartoon illustrating the behaviour of a volcanic plume rising through a stratified ambient (see §4). (*a*) A mixture of buoyant fluid and heavy particles is released at the source. Particles have a relatively small settling speed and so they are transported upwards by the plume; (*b*) upon reaching the neutral buoyancy level, the mixture of particles and plume fluid spreads radially; (*c*) particles separate from the intruding fluid and settle; and (*d*) the remaining fluid is now depleted of particles and rises to its new neutral buoyancy level, forming a higher, anvil-shaped intrusion. (Online version in colour.)

In a volcanic plume, the mixture of hot ash and gas emitted from a volcano entrains and heats the air and within a few kilometres of the volcanic vent produces a buoyant plume of hot air laden with particles (figure 8*a*, cf. [9,32]). As this plume continues to rise, it entrains air at progressively greater heights in the atmosphere, and eventually enters the stably stratified stratosphere. Subsequently, the plume becomes depleted of buoyancy flux as it rises into progressively more stably stratified air, and eventually intrudes to form a spreading ash cloud (figure 8*b*). This ash cloud has a cargo of relatively warm air and dense particles as it spreads laterally at its neutral buoyancy height [20]. In a manner analogous to the pure particle plumes we described above, we expect these particle-laden intrusions to sediment particles as they spread (figure 8*c*), leading to an increase in the buoyancy of the remaining fluid, and a rise in the height of the intrusion relative to a pure thermally driven plume with the same source buoyancy flux (figure 8*d*). Indeed in observing volcanic clouds, there is often somewhat of an up–down asymmetry in the cross-section of the spreading neutral cloud, which has an anvil shape and this may be associated with the separation of some of the particle load (figure 2).

In order to explore this effect, we first present a series of new experiments in which we demonstrate the difference between a pure single-phase buoyancy-driven plume, and a two-phase plume, in which the particles settle from the plume. We then develop a simple model for the increase in ascent height, at least at early times, and consider the implications of this for assessing the mass flux in volcanic plumes.

### (a) Volcanic plumes: experiments

In the experiments modelling a volcanic plume, we released fresh water laden with particles into a stably stratified aqueous solution. The source fluid had a sufficiently small particle load that it was less dense than the environment at the base of the tank, and the mixture therefore rises to form a buoyant plume. On reaching its neutral height, the fluid–particle overshoots a small amount and then falls back to spread out at the neutral height. However, as the particles begin to separate from the radially spreading intrusion, the residual fluid becomes more buoyant and rises upwards, forming an asymmetrical anvil-type neutral cloud, somewhat reminiscent of the volcanic clouds seen in figure 2.

**Figure 9.**
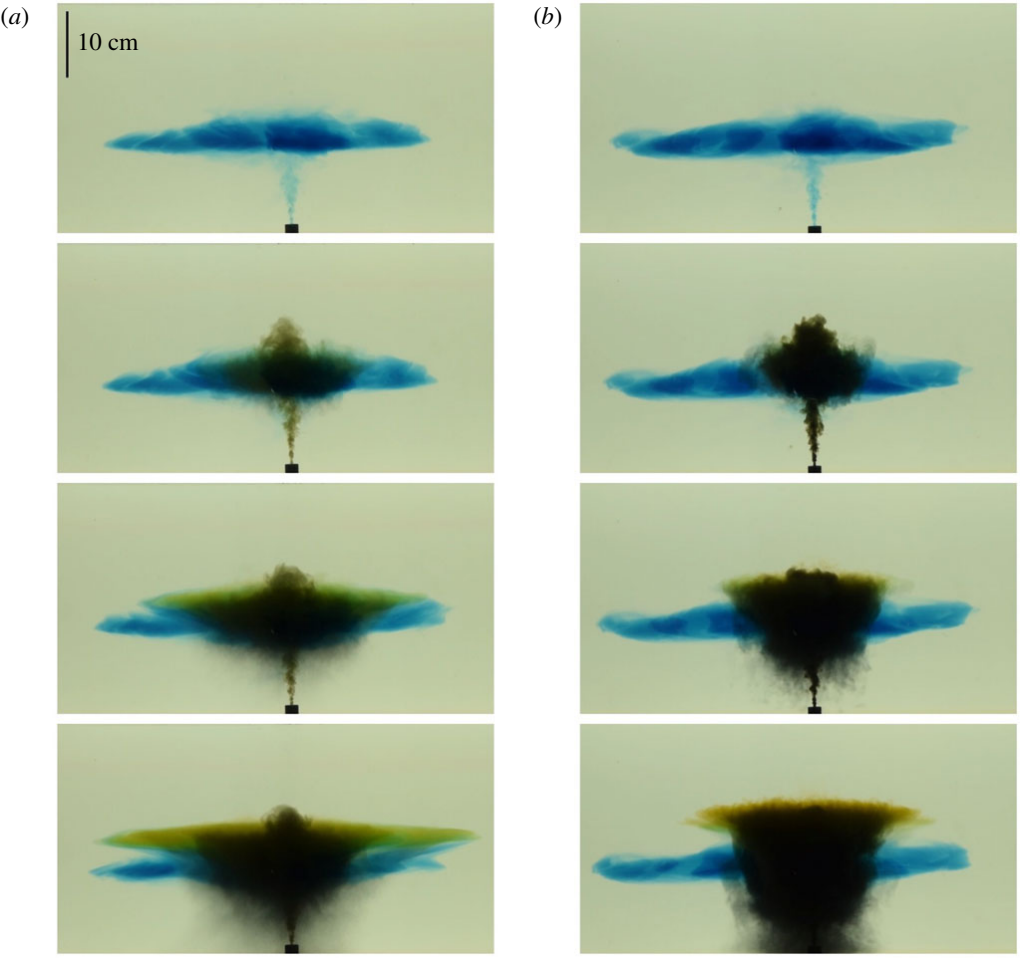
Four photographs captured at successive times during two particle plume experiments (experiments q and t, see table 3). We compare the height of rise and intrusion dynamics of two model volcanic plumes, in which a mixture of green low-salinity fluid and particles rises through a continuously stratified aqueous solution. The particle concentration in experiment (*a*) has a negative buoyancy equal to a fraction 1/6 of the positive buoyancy associated with the salinity of the source fluid, while in experiment (*b*) it has a negative buoyancy equal to a fraction 5/6 of the positive buoyancy associated with the salinity of the source fluid. However, the bulk buoyancy of the particle-fluid mixture at the source is equal in the two experiments. In each experiment, the particle-laden fluid has the same initial bulk buoyancy and flow rate as the purely blue saline fluid which was released earlier and led to formation of the laterally spreading blue intrusion. The initial height of each particle-laden plume is the same as the blue plume; however, as particles sediment from the top of the plume, the remaining fluid becomes less dense, and so it rises to the level of the green intrusion. This leads to an anvil-type shape which is reminiscent of the neutral clouds seen at several volcanoes as shown in figure 2. It is seen that the green intrusion in experiment (*b*) develops at a larger distance from the source than that in experiment (*a*), owing to the difference in particle concentration between the two experiments. As each experimental system evolves, some particles are re-entrained into the plume, and the dynamics becomes more complex. The photographs depicted in (*a*) were captured at times 2s, 33s, 76s and 128s after the beginning of the experiment, while those depicted in (*b*) were captured at times 2s, 35s, 63s and 117s after the beginning of the experiment. (Online version in colour.)

**Figure 10.**
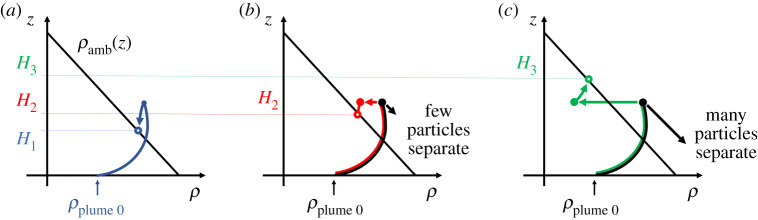
Illustration of how the sedimentation of particles in the neutral cloud leads to an increase in the buoyancy of the remaining fluid and an increase in the final intrusion height. In (*b*), the particle load is modest, so the change in buoyancy is small. As a result, the particle plume fluid intrudes at a height which is moderately larger than that of a single-phase plume with identical buoyancy flux (*a*). In (*c*), the particle load is higher, with a corresponding reduction in the salinity of the source fluid, so that the net buoyancy of the source is the same. However, the change in the buoyancy in the neutral cloud is larger and the intrusion eventually develops at a considerably greater height than the initial plume. (Online version in colour.)

In figure 9, we compare the height of an intrusion from a single-phase plume, shown in blue, with the green intrusions of the fluid in two particle-laden plumes which have the same net buoyancy as the blue fluid at the source. It is seen that initially the green intrusions exactly overlap the blue intrusion, as expected, but as the fluid spreads radially, particles fall out of the neutral cloud, and the green intrusions rise. We also note that, as expected, the impact of particle fallout is larger when the concentration of particles in the plume fluid is larger (figure 9*b*), leading to a higher intrusion of the green fluid (figure 10).

### (b) Volcanic plumes: model of the increase in the intrusion height

The initial buoyancy at the source is given by

4.1 $$g_{o}^{\prime }=g_{f}^{\prime }-g_{p}^{\prime }=\left(\frac{\rho_{f}-\rho_{\text{amb}}}{\rho_{\text{amb}}}-\frac{\rho_{p}-\rho_{\text{amb}}}{\rho_{\text{amb}}}C\right)g,$$

where subscript *f* and *p* denote fresh water and particles, respectively, and where *ρ*_amb_ is the density of the ambient fluid at the level of the source (see figure 10), while *C* is the concentration of particles in the source fluid (cf. [33], see tables 1–3). The simple argument given earlier in the paper suggests that to good approximation, at the neutral buoyancy height, the diluted source fluid has a buoyancy 0.22*N*^2^ *H*, which corresponds to the fluid at a height of *H*_*o*_ = 0.22*H* of the total plume height. Writing *H* in terms of the stratification and buoyancy flux we obtain the result

4.2 $$H_{o}=1.1\pi^{-1/4}B_{0}^{1/4}N^{-3/4}.$$

If the particles fall from the fluid in the intrusion, this component of the buoyancy increases by the fraction

4.3 $$\frac{g_{p}^{\prime }}{g_{f}^{\prime }-g_{p}^{\prime }},$$

and this leads to a corresponding increase in the height by the amount

4.4 $$\Delta H=\frac{g_{p}^{\prime }}{g_{f}^{\prime }-g_{p}^{\prime }}H_{o}.$$

We note that this estimate of the increase in the height of the intrusion associated with the sedimentation neglects other effects, such as the eventual re-entrainment of some of the sedimenting particles as the particles fall past the main ascending plume. This can affect the plume dynamics, since these re-entrained particles reduce the buoyancy of the plume, and may then reduce the plume height or even cause plume collapse [11,21,23,26]. However, the time for this re-entrainment to become established is longer than the time for the fluid in the initial phases of the intrusion formation to sediment its particles and re-adjust to a new neutral height, and we have measured this new neutral height by tracking the appearance and radial spreading of the particle-free green fluid in the intrusion, as may be seen in the final panels of figure 7. The subsequent evolution of the system becomes more complex owing to the re-entrainment.

**Figure 11.**
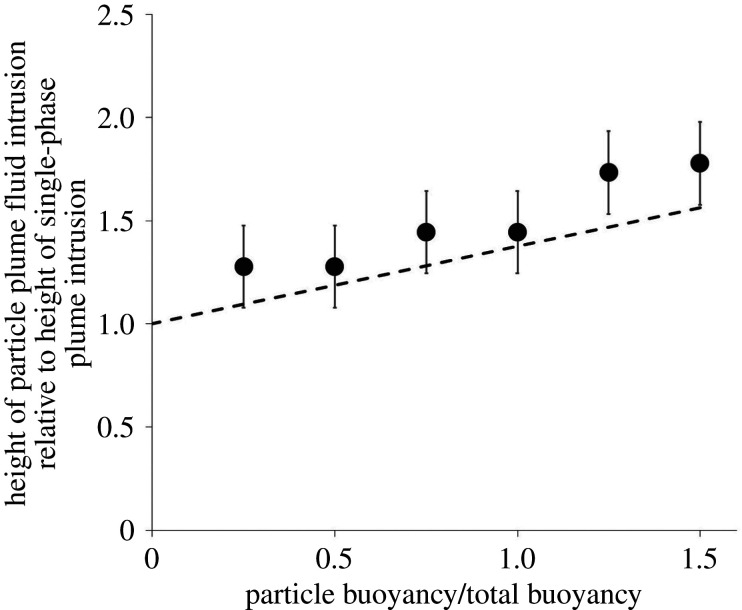
Data illustrating the initial height of the intruding fluid, following the sedimentation of the particles, as a fraction of the height of the equivalent pure fresh water plume (experiments p–u, see table 3). In each experiment, the source fluid had the same net buoyancy, but the salt content and the particle load of the source fluid were both changed from experiment to experiment. We compare the measurements of the height of the intrusion with the above relation for the height, *H*_*o*_ + Δ*H*, and obtain reasonable agreement.

In figure 11, we present our experimental data illustrating the height of this initial intruding fluid, following the sedimentation of the particles, as a fraction of the height of the equivalent pure fresh water plume (see figure 9 for two example experiments). In each experiment, the source fluid had the same net buoyancy, but the salt content and the particle load of the source fluid were both changed from experiment to experiment in order to amplify the effect of the sedimentation on the intrusion height (cf. Figure 10). We compare the measurements of the height of the intrusion with the above relation for the height, *H*_*o*_ + Δ*H* (equation 4.4), and obtain reasonable agreement. For reference in these experiments, the net buoyancy of the source fluid was *g*′ = 0.725 m s^−2^, the buoyancy flux *B*_0_ = 2.9 × 10^−6^ m^4^ s^−3^ and the stratification in the ambient was characterized by *N* = 0.729 s^−1^ (table 3).

In assessing the height of rise of a volcanic cloud, the driving force for the eruption column is given by the buoyancy associated with the thermal energy minus the buoyancy associated with the ash particles in the cloud. A common practice has been to estimate the eruption rate using the theory of turbulent buoyant plumes, in which the thermal energy of the erupting material generates the buoyancy through heating ambient air, and then in which the ambient stratification arrests the ascent of the plume [9]. To this end, if we take the thermal energy flux of the hot ash, and use this to calculate the buoyancy flux in a plume of warm air laden with the ash particles, we can find an estimate for the buoyancy flux of the plume. Heat conservation leads to the relation

4.5 $$Q_{m}\rho_{m}C_{p_{m}}(T_{m}-T_{o})=Q_{a}\rho_{a}C_{p_{a}}(T_{a}-T_{o})=\left(Q_{a}\frac{T_{a}-T_{o}}{T_{o}}g\right)\frac{\rho_{a}C_{p_{a}}T_{o}}{g},$$

where *ρ* is the density, *C*_*p*_ the heat capacity and *T* the temperature, and where subscripts *a* and *m* denote the air and the particles in the plume, respectively, while *T*_*o*_ is the reference temperature of the ambient air outside of the plume. In equation (4.5), the thermal buoyancy of the air plume is

4.6 $$B_{T}=Q_{a}g_{T}^{\prime }=Q_{a}\frac{T_{a}-T_{o}}{T_{o}}g.$$

The particle load has mass flux *Q*_*m*_*ρ*_*m*_ and this is distributed in the warm air, with mass flux *Q*_*a*_*ρ*_*a*_, leading to a negative particle-related buoyancy

4.7 $$g_{p}^{\prime }=g\frac{(\rho_{m}-\rho_{o})Q_{m}}{\rho_{o}Q_{a}},$$

and hence a particle buoyancy flux given approximately by the relation

4.8 $$B_{p}=-gQ_{m}\frac{\rho_{m}}{\rho_{o}}.$$

Assuming the particles are carried upwards by the plume, the height of rise of the plume is then given by the classical relation (1.3), where *B* is the net buoyancy

4.9 $$B=B_{T}+B_{p}.$$

Given the above modelling of plumes in which we find that the buoyancy of the source fluid combined with the dilution through mixing represents vertical displacement by a distance of 0.22 *H*, then if the particles settle from the laterally spreading intrusion, that fraction of the buoyancy is lost, leading to an increase in the height by (cf. equation (4.4))

4.10 $$\Delta H=0.22H\frac{g_{p}^{\prime }}{g_{T}^{\prime }-g_{p}^{\prime }}.$$

Using the definitions of the particle and thermal buoyancy above, we can reduce this relation to the simpler form

4.11 $$\Delta H=0.22H{\left[\frac{(T_{m}-T_{o})C_{p_{m}}}{T_{o}C_{p_{a}}}-1\right]}^{-1}.$$

**Figure 12.**
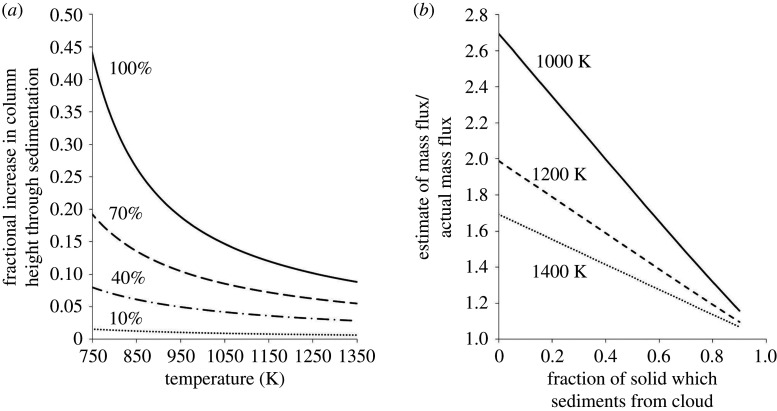
(*a*) Fractional increase in height Δ*H*/*H* as a function of the magma temperature, in the case that 10, 40, 70 and 100% of the particles sediment from the ash cloud as it spreads laterally. (*b*) Range of possible buoyancy fluxes as a function of the fraction of solids which sediment and of the initial temperature of the erupting material.

In figure 12*a*, we show the fractional increase in height Δ*H*/*H* as a function of the magma temperature, in the hypothetical cases that 10, 40, 70 and 100% of the particles sediment from the ash cloud as it spreads laterally. This sedimentation increases the buoyancy of the remaining air-particle suspension, leading to this increase in the height. The result shows that the height of the spreading intrusion is strongly influenced by both the thermal energy flux and the particle flux produced by the eruption, as well as the fraction of these particles that separate from the intrusion as it spreads radially. The increase in the buoyancy will tend to produce the anvil-shaped cloud seen in both the laboratory and the field observations.

Many field observations suggest that the fraction of particles which separate from the intrusion in the near field may be in the range 10–40% (cf. [9]). Very fine fractions of ash may have a small settling speed and thereby require a greater distance before they settle from the intrusion (cf. [10,23–25]). As illustrated in figure 12, if 10–40% of the material sediments from the plume, we expect that the column height may increase by approximately 3–8% of the total plume height. Hence, for volcanic plumes that rise 10–20 km above the vent, particle sedimentation from the spreading intrusion may result in an increased height of order 0.5–1.5 km, which is consistent with the anvil type deformation seen in figure 2.

One interesting implication of this result (figure 12*a*) is that if one attempts to estimate the total buoyancy flux of an eruption column based on the relation with the height of rise of the ash plume, then there is some uncertainty depending on the fraction of the particles which sediment from the top of the plume as the cloud spreads laterally. The conventional approach is to take the height of rise of the ash plume, and use the relation (1.3) to relate the thermal buoyancy flux *B*_*T*_ with the height of rise, neglecting the effect of the particles [9]. However, if we relate the net buoyancy flux, *B*_*T*_ + *B*_*p*_, to the height of rise, as described in the model above (equation (1.3)), and then estimate the additional height of rise owing to the sedimentation of the particles, as shown in figure 12*a*, we find that for a given column height there is a range of possible values for the buoyancy flux. In figure 12*b*, we illustrate this range of values in terms of the fraction of solids which sediment and the initial temperature of the erupting material, normalizing the results relative to the hypothetical reference case in which all of the particles sediment. It is seen that if we assume that 10–40% of the particles sediment from the spreading cloud, then for a given height of rise the predicted thermal energy flux is about 5–20% smaller than the case in which we assume no particles sediment, since the larger the particle load the larger the thermal buoyancy required to overcome the negative buoyancy of the dense particles.

## 5. Conclusion

Experiments and simplified models have been used to describe the motion of the fluid and the particles in turbulent particle-laden plumes. Cases in which the particles provide the buoyancy driving the plume, as relevant for deep-sea mining, and in which the particles reduce the buoyancy driving the plume, as relevant for volcanic plumes, have been considered. We have demonstrated that even if the particle fall speed is much smaller than the characteristic speed of the plume, so that the particles are carried with the plume, the subsequent sedimentation of the particles can have a leading order impact on the height of rise of the fluid carried by the plume. This is key for assessing the potential environmental footprint of particle plumes produced during deep-sea mining, and also for assessment of the eruption rate from measurements of volcanic eruption columns and the associated laterally intruding umbrella clouds. The process of sedimentation also leads to radially spreading intrusions of plume fluid which have an asymmetric shape reminiscent of that seen in volcanic eruption columns.

We should emphasize that the modelling in this paper is highly simplified, and aims to explore the physical controls on the system. It will be interesting in the future to develop this approach and explore some of the key additional effects which can influence or modify the process, including the effects of wind [13,19] and the effects of the grain size distribution and the aggregation of ash in the spreading cloud [9].

In conclusion, we note that it would be interesting to carry out further experiments to explore some of the processes controlling the sedimentation of particles falling from the spreading intrusion in terms of the possible convective sedimentation and re-entrainment of the particles into the plume [3,14,23,26,29,30], and the impact of these processes on the long-term evolution of the intrusion and the plume.

## Data accessibility

This article has no additional data.
